# Photographed Rapid HIV Test Results Pilot Novel Quality Assessment and Training Schemes

**DOI:** 10.1371/journal.pone.0018294

**Published:** 2011-03-31

**Authors:** Yu-Ho C. Chiu, Joanna Ong, Sandy Walker, July Kumalawati, Tintin Gartinah, Dale A. McPhee, Elizabeth M. Dax

**Affiliations:** 1 NRL, St Vincent's Institute, Melbourne, Victoria, Australia; 2 Clinical Pathology Department, Medical Faculty, University of Indonesia, Dr Cipto Mangunkusumo Hospital, Jakarta, Indonesia; 3 West Java Provincial Health Laboratory, West Java Health Office, Bandung, Indonesia; 4 Department of Microbiology and Immunology, University of Melbourne, Parkville, Victoria, Australia; University of California, San Francisco, United States of America

## Abstract

HIV rapid diagnostic tests (RDTs) are now used widely in non-laboratory settings by non-laboratory-trained operators. Quality assurance programmes are essential in ensuring the quality of HIV RDT outcomes. However, there is no cost-effective means of supplying the many operators of RDTs with suitable quality assurance schemes. Therefore, it was examined whether photograph-based RDT results could be used and correctly interpreted in the non-laboratory setting. Further it was investigated if a single training session improved the interpretation skills of RDT operators. The photographs were interpreted, a 10-minute tutorial given and then a second interpretation session was held. It was established that the results could be read with accuracy. The participants (n = 75) with a range of skills interpreted results (>80% concordance with reference results) from a panel of 10 samples (three negative and seven positive) using four RDTs. Differences in accuracy of interpretation before and after the tutorial were marked in some cases. Training was more effective for improving the accurate interpretation of more complex results, e.g. results with faint test lines or for multiple test lines, and especially for improving interpretation skills of inexperienced participants. It was demonstrated that interpretation of RDTs was improved using photographed results allied to a 10-minute training session. It is anticipated that this method could be used for training but also for quality assessment of RDT operators without access to conventional quality assurance or training schemes requiring wet samples.

## Introduction

Classic testing strategies for HIV diagnosis that yield close to 100% predictive values, involve testing a plasma or serum sample with an enzyme immunoassay followed by Western blot [1]. These strategies require dedicated laboratory equipment and experienced laboratory-trained operators. The reporting times are unsatisfactory and may take up to 2 weeks for a positive result to be delivered [2]. With the extensive roll-out of anti-HIV therapies in the last 5 years, faster and more accessible testing has become imperative. Furthermore, it is preferred by public health groups in the US where the rate of individuals returning for their results has been low in some situations [3]. Therefore, to ensure readily available and effective HIV diagnosis, decentralized testing employing rapid diagnostic tests (RDTs) has been widened considerably. RDTs that can be performed and interpreted reliably by non-laboratory-trained operators in non-laboratory settings are being used extensively. Testing in facilities such as voluntary HIV counseling and testing centers (VCT) and venues such as bathhouses has become the norm [4], [5].

HIV RDTs are immunoassays that are simple to perform, give results without the need for laboratory equipment, are accurate and can provide on-site (negative) results with a single visit [4], [5], [6], [7], [8]. Their use resolves several problems in HIV diagnosis especially in remote or resource-limited settings. For example, individuals who cannot get to a laboratory facility may be reached by providing more widespread settings for testing by non-laboratory trained personnel. The burden on centralized testing facilities, again especially in resource-limited settings, may allow individuals to obtain their test results (at least negative test results) in a single visit. Therefore, in 2009 RDTs were widely used in non-laboratory settings by operators with limited or no laboratory experience [7]. However, RDTs are immunoassays which may be fraught with difficulties including those associated with accurate interpretation. The interpretation of their results is subjective. Often, instructions from test manufacturers illustrate schematically, examples of strong results along with strong intensity control and test lines/dots. Operators without extensive training and who may themselves, be trained by non-laboratory based personnel, misunderstand that positive RDT results may be weakly reactive in some cases. Furthermore, considering false reactivity as a possible result is sometimes discouraged actively. Such cases are often misinterpreted as anti-HIV negative. The concept of false reactivity is also not well understood in numbers of testing systems in Asia. In addition, the number of different RDTs available in some countries may be extremely high. In one country 80 different tests were found to be in circulation by the authors. In some cases the same test is rebranded so that two seemingly different tests may actually be the same test. Therefore, quality assurance programmes are necessary to maintain the quality of testing outside laboratories, just as they are in laboratories. With the large variety of HIV RDTs used in large numbers of settings by operators with different levels of laboratory experience, the logistics of presenting adequate, or any, quality programmes presents serious difficulties.

Provision of External Quality Assessment Schemes (EQAS) is one approach to assess potential errors or difficulties. Classically, the EQAS approach assesses the ability of a facility to perform all steps of the testing process - from sample reception to results' reporting - and may assess if the correct testing outcome (i.e. reactive or non-reactive) is delivered. Because HIV RDTs are so widely used (especially in remote settings), implementation of classical EQAS is difficult and impractical for both providers and participants. For EQAS providers, it may be almost impossible to collect adequate quantity of sera or plasma with known HIV status to generate a single panel for every testing facility. Equally well, the logistics of distributing EQAS panels while maintaining the quality of samples, e.g. without a cold chain in some settings, may also be difficult. In addition, samples might show different reactivity to different RDTs. For example, a sample might show a weakly reactive result in a screening test in one laboratory or testing facility and negative result in a different screening test in another. The EQAS provider needs to ensure the sample panel would produce matching results in the screening and confirmatory RDTs employed by all testing facilities. This may add to the problem of collecting adequate quantities of sample for any given EQAS exercise. Clearly, other approaches to monitor quality should be welcomed. A medium for assessment that can be distributed widely in a cost-effective manner would be a more practical way to improve the quality of HIV testing using RDTs.

We have performed a proof of concept study using photographed RDT results [9]. We postulated that photographed results of RDTs could be used as a cost-effective method of EQAS. Using photographed RDT results showed that experienced and inexperienced readers delivered correct interpretations of the photographed results. However, the photographs used in our initial study were not captured under carefully controlled conditions and settings such as lighting, exposure and color balance varied. Quality photographic results could be distributed with consistency and reproducibility if a controlled and standardized approach was controlled.

In the present study the assessment of photographed results was found to be consistent and reproducible. Photographed results of panels of 10 samples assayed in four HIV RDTs were used to assess and compare the proficiency of interpretation by three groups of operators (non-laboratory trained personnel, laboratory trained operators and a group of pathologists attending a national meeting). Their ability to interpret the photographed results and their responses to a short tutorial on interpretation were compared. All participants had performed RDTs, at least once and they may or may not have been the RDTs included in this training. The photographed results, on formatted sheets, were presented in two sessions with a 10-minute tutorial on the subtleties of interpretation after the first session. The aims were i) to investigate the usefulness of photographed results for assessing RDT operator proficiency to interpret results, ii) to determine if training improved the proficiency of operators' ability to interpret results and iii) to assess underlying factors for both RDTs and operators that might contribute to a lack of ability to correctly interpret RDT results before training. RDT results presenting faint lines and/or requiring differentiation of HIV-1 from HIV-2 results were selected to present more difficult results for interpretation. Operators who had not performed RDTs included in this training showed the most improvement in interpretation after the brief tutorial suggesting that the technique could be used not only as a quality assurance measure, but as a training tool.

## Materials and Methods

### HIV rapid diagnostic tests

The RDTs used in this study employed technologies such as lateral flow and flow-through immunochromatography. These included **Advance Quality** HIV Rapid Test (In Tec Products, Xiamen, China), **Determine** HIV-1/2 (Inverness Medical, Princeton, NJ, USA), **Insti** HIV-1/HIV-2 (BioLytical Laboratories, Richmond, Canada) and **SD Bioline** 1/2 3.0 (Standard Diagnostics Inc, Kyonggi-do, Korea). The RDTs were chosen so that the interpretation was either two-line/dot for both control and HIV-1/HIV-2 indicators (Advance Quality, Insti and Determine) or three-line RDT which presents lines for control, HIV-1 and HIV-2 indicators (SD Bioline).

### Preparation of the Photographed panel of RDT Results

Because DSLR cameras allow full manual control to accommodate different environments and RDTs, it was chosen over the compact digital cameras to accurately reflected the colour, contrast and the intensity of lines on the RDTs, thus reproducing the actual RDT results as seen by the readers of the tests' results. Photographs of RDTs showing typical strong lines from well-characterized reactive samples by the NRL (Melbourne, Australia) were imported as a layer in GNU Image Manipulation Program (GIMP, open source). This layer was then duplicated and both layers were renamed as control layer and test layer. The control line in the control layer and the test line in the test layer were cropped and remained in position. A photograph of a blank RDT was imported as a background layer and saved as a master file. By adjusting the opacity of the control and test layer from 100 to 75, 50, 25, 10, 5 or 0 percent opacity, a range of possible results were readily available to be exported to JPEG format. Images were then imported to a word processor program as a photographed panel for printing. Computer monitor and printer were calibrated by ColorMunki Photo (X-Rite Inc, USA) to ensure printouts accurately reflected the RDT results shown on the computer monitor.

### Validation and Classification of the Photographed Results

Laboratory-trained staff who had performed and interpreted results for the four RDTs ensured the line patterns and the shapes of control and test lines were the same as results from using actual samples. The photographer with no laboratory experience who had previously photographed the RDT results ensured that the color and contrast of lines were similar to the actual RDT results. Both laboratory trained staff and photographer were able to identify the faint lines and deliver correct results. “Obvious” and “Difficult” results were included in the panel of 10 photographs for each RDT. While “obvious” results were those with clearly visible lines with patterns shown in the manufacturers' instruction, “difficult” results were those that required the identification of a faint line and/or the comparison of intensity for HIV-1 and HIV-2 lines.

### Participants

The study was conducted in Indonesia at an annual pathologists' meeting held in Jakarta and at a training session held in Bandung, West Java Province. Participants included staff with and without laboratory experience, as well as staff with authority to interpret and adjudicate RDT results. There were three groups of participants: non-laboratory trained staff of voluntary counseling and testing centers (n = 19), laboratory-trained and -based staff of provincial health laboratories in Surabaya, Jakarta, Bandung and West Papua (n = 22), and pathologists (n = 34). All agreed to participate. A questionnaire was designed to collect information on the four RDTs in this study and other RDTs such as i) levels of experience; ii) previous training; iii) access to kit inserts and other background information of participants such as areas of expertise. Participants were defined as experienced for a RDT if they had performed a test at least once.

### Training

There were two sessions when the photographed test results were read: before training and after training. Photographs for both sessions were identical, but in different order. In each session, participants interpreted the 10 photographed results for each of the RDTs. They were then asked not to correct their interpretation and were given the correct interpretation for each sample. Questions were allowed and were answered frankly. During the training, each participant received simplified interpretation instructions and a 10-minute tutorial to explain the interpretation instructions for each of the RDTs. Following the training session, participants were encouraged to discuss their results with their peers in order to emphasize the importance of consultation in interpreting RDT results.

### Statistical Analyses

The known opacity values for control and test lines/dots were used to establish the reference result for every photograph. All reported results were transcribed into a spreadsheet, and checked by independent personnel to ensure no transcription error. Illegible reported results were excluded. Reported results were compared with the reference results, and categorized as correct or incorrect. SPSS 11.0 was used to perform non-parametric Chi-Square test in order to determine the probabilities of difference between variables.

### Ethics Statement

Verbal consent was obtained from all participants in terms of their agreement to participate in the study. Quality assurance is exempted from Human Research Ethics Committee approval by St. Vincent's Hospital, Melbourne. Ethics approval was obtained for testing the original samples from St Vincent's Health, Protocol No.: HREC-A 079/05. All the original reference samples were obtained anonymously under the protocol used and all data were analyzed anonymously.

## Results

**Figure 1:**
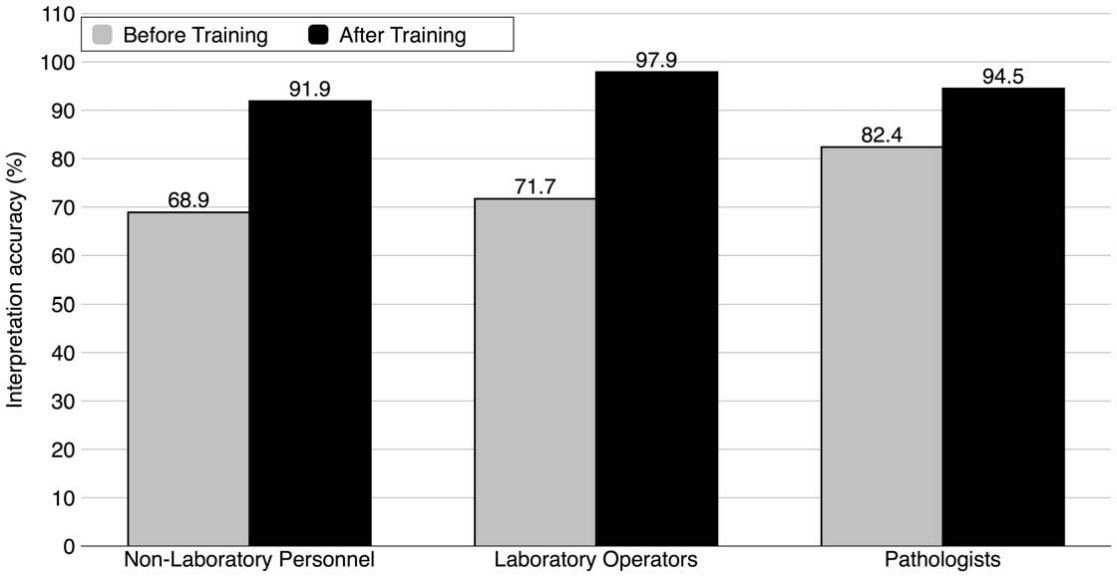
Overall improvement in the interpretation accuracy for the three groups of participants. Non-laboratory based personnel (n = 19), laboratory based and trained technical staff (n = 22) and a group of pathologists attending a national meeting (n = 34) interpreted photographed results of 10 samples in four rapid diagnostic tests (RDT, see Methods Section). Each participant read 10 results for each test then was given an ∼10 minute tutorial on how to read the tests accurately and each was supplied with manufacturers' instructions. Training improved interpretation accuracy for each participant group (non-parametric Chi-Square test, p<0.05 for all groups).

**Figure 2:**
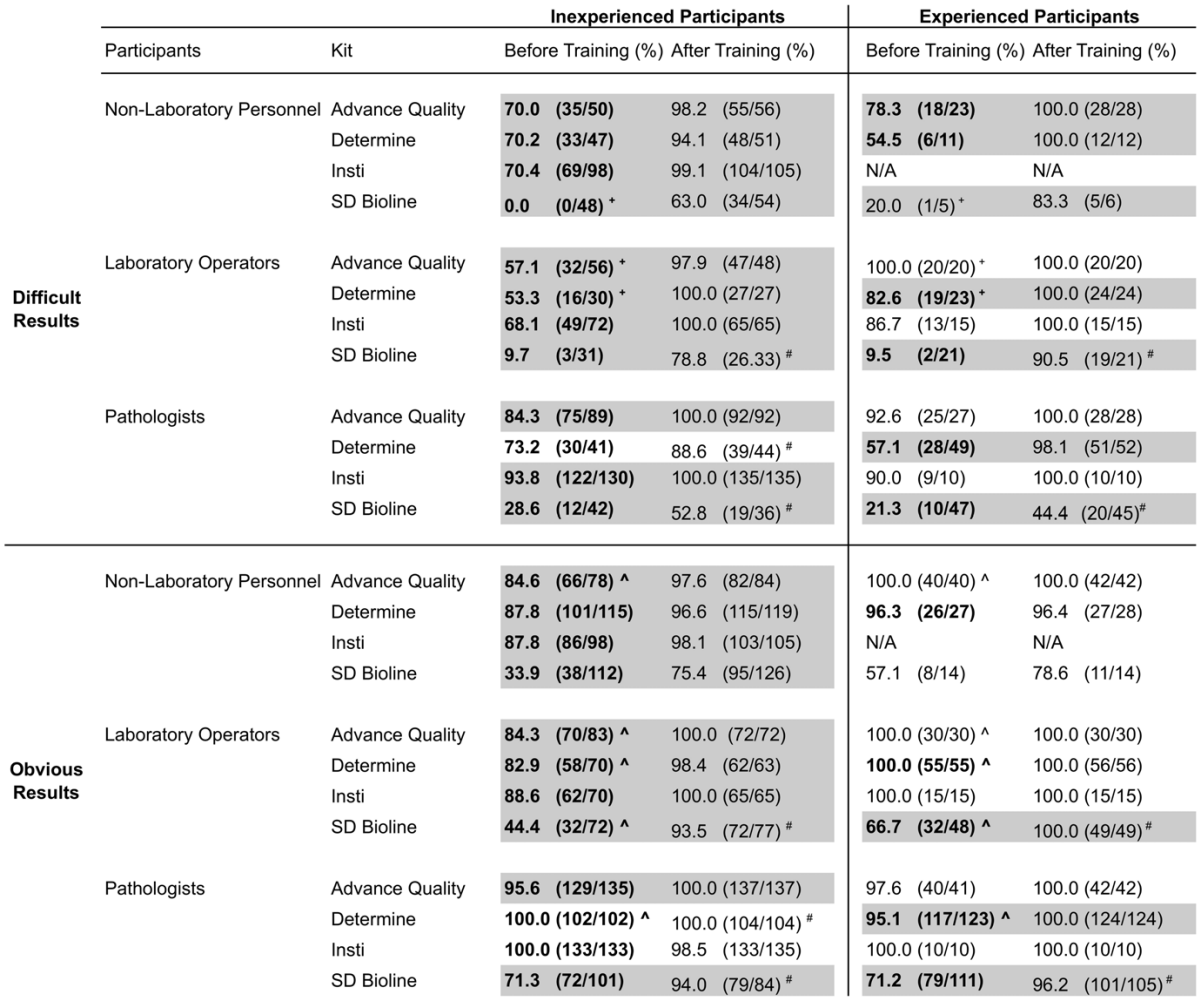
Interpretation accuracy of four rapid diagnostic test (RDT) kits by three groups of participants before and after training. The table is divided into four quadrants by the level of participants' experience (inexperienced/experienced) and the difficulty of the RDT results (obvious/difficult). p<0.05 from non-parametric Chi Square analysis is considered as significant difference. Grey block indicated the significant differences between before and after training. **Bolded numbers** indicated significant differences between difficult and obvious results before training. # indicated significant differences between difficult and obvious results after training. ∧ indicated significant differences between inexperienced and experienced participants for obvious results. + indicated significant differences between inexperienced and experienced participants for difficult results.

**Figure 3:**
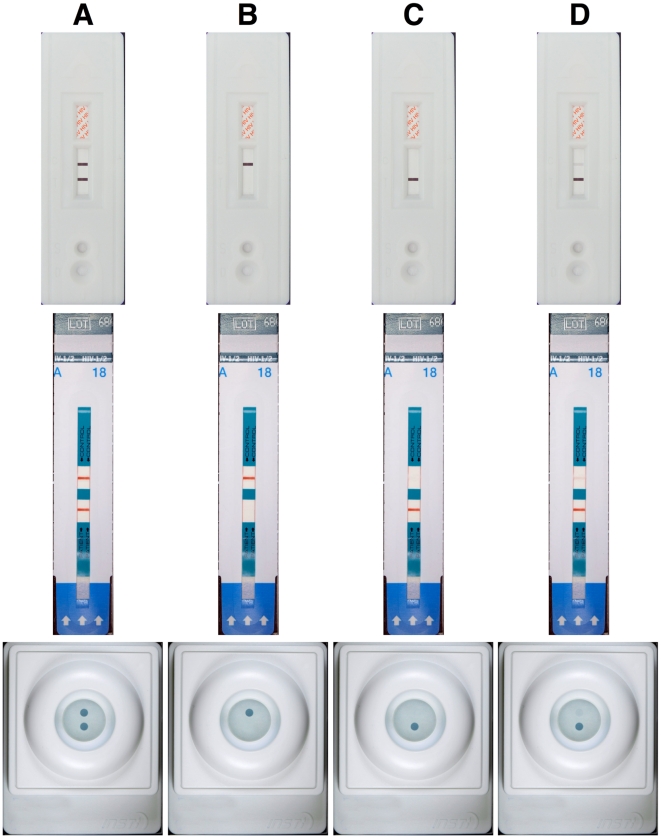
Possible types of result for two-line/dot HIV rapid diagnostic tests (RDTs). RDTs from top to bottom are: Advance Quality, Determine and Insti. Typical results for reactive (A), non-reactive (B) and invalid sample results (C) as shown in manufacturers' instruction were considered "obvious". The results labeled D were considered "difficult" because of the presence of only a faint line/dot and because both lines/dots are not the same intensity.

**Figure 4:**
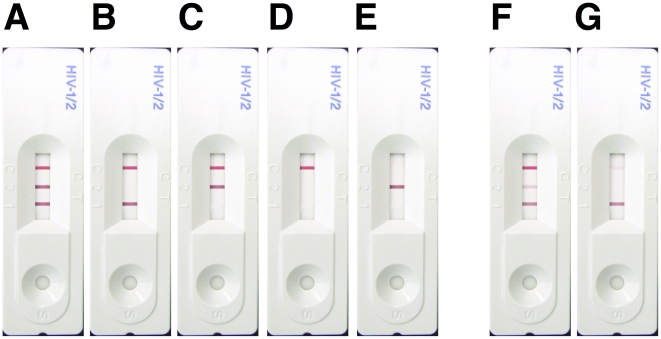
Possible results for the three-line SD Bioline HIV rapid diagnostic test (RDT) that presents indicator lines for control, HIV-2 and HIV-1. HIV-1/2 positive (A), HIV-1 positive results (B, F & G), HIV-2 positive (C), HIV negative (D) and Invalid (E) results. A-E were considered "obvious" because the manufacturers' instruction illustrated these types of result. F & G are both HIV-1 positive and considered "difficult" because the correct interpretation required comparison of intensities for the middle and bottom lines (F), or the ability to see and interpret a faint line (top line, G).

Training using photographed RDTs was effective in improving the interpretation accuracy of RDTs. Participants exhibited an overall improvement in interpretation after training (75.8 vs 94.6%, p<0.05) shown in Figure 1. Figure 2 shows further analysis of the improvement in interpretation into four quadrants by the experience of the participants and the difficulty of the results. Typical obvious and difficult results for two-line/dot RDT and three-line RDT are shown in Figures 3 and 4, respectively. Experienced participants showed significant improvement upon training in more participant/kit combinations for difficult results than the obvious results (indicated in grey blocks in Figure 2). Similarly, inexperienced participants showed significant improvement upon training in more participant/kit combinations than the experienced participants (indicated in grey blocks in Figure 2), regardless of the difficulty of the results. Less improvement upon training for experienced participants was because the accuracy was already high (≥86%) before training (except for non-laboratory personnel reading the SD Bioline results, see Figure 2). The most dramatic improvements in correct interpretations were observed for inexperienced participants with the difficult results (Top-left quadrant, Figure 2). There were some instances where no improvement in interpretation was demonstrated following training of the participants either because they could not be improved upon (100%) or there were no data for some combinations of experience and difficulty of results (e.g. none of the non-laboratory operators had experience with Insti).

We analyzed the data obtained before training to assess the interpretation accuracy of participants. Obvious results were interpreted with higher accuracy than the difficult results for inexperienced and experienced participants in 12 and 6 of the 12 participant/kit combinations (indicated in **bolded** numbers in Figure 2), respectively. Likewise, experienced participants interpreted RDTs with significantly higher accuracy than their inexperienced counterparts in three (indicated in “+” in Figure 2) and four (indicated in “∧” in Figure 2) of the 12 participant/kit combinations, respectively. The exception was the experienced pathologists reading the obvious Determine test results less accurately than the inexperienced pathologists (95.1 vs 100.0%, p<0.05).

We used the data obtained after training to assess the effectiveness of the interpretation training by photographed RDTs. Regardless of the difficulty of the results, there was no significant difference in interpretation accuracy between experienced and inexperienced participants, indicating training helped to minimize the gap between inexperienced and experienced participants to interpret correct results. However, regardless of the level of experience, laboratory operators and pathologists showed significantly lower accuracy in interpreting the difficult results than the obvious results for SD Bioline (indicated in “#” in Figure 2). Therefore, a more concentrated training effort possibly with a tailor-made photograph panel focusing on difficult results of SD Bioline may be necessary if this test were to be used. Of note, the results of the reading of SD Bioline results consistently showed the lowest accuracy in all participant/kit combinations, presumably because of the unfamiliar 3-line format and the requirement to differentiate HIV-1 from HIV-2 signals.

## Discussion

The use of photographed RDT results is an objective means of assessing the interpretation proficiency of RDT operators. The results of the present study demonstrated that experience and proficiency were not necessarily equivalent. The results did provide evidence that proficiency depends on experience and suggests that experience and training are important before proficiency can be assured.

It was found that brief, targeted training can in some circumstances dramatically improved interpretation of RDT results for inexperienced participants (and experienced participants to lesser extent) and photographed results could be used for training and interpretation instead of running actual samples on actual RDTs. The most dramatic improvements were observed for interpreting the SD Bioline assay's results by inexperienced participants. The photographed RDT results along with the information sought from all participants helped to identify factors that contributed to better interpretation. While inexperienced participants showed significantly improved interpretation upon training in almost all circumstances, experienced participants showed significant improvement again if the results are more difficult to interpret. The results in the present study indicated the need for periodic retraining programs for personnel using RDTs.

Interpretation of difficult results offered opportunity for the most effective training. The results also indicated that inexperience in interpretation of results was readily detected by this method and could be remedied with retraining among the RDTs tested [10].

Publications indicated that many different types of RDTs can be performed and read with accuracy [4], [6], [7], [11], [12], [13]. Counselors or laboratory technologists are trained by following guidelines such as those provided by WHO and CDC [14]. However, there have been high levels of false positive results reported using RDTs [10], [15]. Klarkowski et al reported that despite nurse counselors' being trained, false negative results were observed, especially if the results were weakly reactive in these situations.

There are two essential technical aspects involved in implementing photographed RDT results to assess proficiency of interpretation. The photographs must be produced with consistency and reproducibility. As with all EQAS, participants must receive identical samples if valid comparisons between laboratories or in this case, operators are to be made. If such comparisons are to be valid the photographs must be consistent in color, contrast and intensity for the line/dot indicators. While consistency refers to photographs that are generated from computer and printer at one site with identical quality, reproducibility refers to the implementation of the single photographic setting at a different location with identical quality. The present study presented evidence that consistent photographs from a single site could be used as effective training and assessment tools. For other laboratories to adapt and implement photograph-based training, reproducibility is a major consideration. Further work is needed to assure reproducible photographs generated by multiple sites. Reproducibility is commonly achieved in the field of photography and desktop publishing with the help of commercial calibrators for computer monitors and printers.

There are several implications that arise from the results in the present study. First, photographed RDTs can assess the interpretation proficiency of operators in a more cost-effective and widespread manner than the classic EQAS. Because interpretation is the only step of the RDT operation that depends on the HIV status of the sample, operators shown to be proficient in performing the test can be assessed/retrained for interpretation by photographs instead of using the actual characterized samples and RDTs. To establish proficiency in performing the tests *in toto* would not be possible with photograph-based training. Second, photographed RDT results may assist in improving the design and implementation of HIV testing algorithms. Typically, HIV diagnosis relies on a serial strategy of using tests for screening followed by confirmation of a reactive result. The candidate tests are usually selected based on their performance characteristics such as specificity and sensitivity. Effective diagnosis requires correct operation **and** interpretation of the candidate tests [7]. However, lower accuracy of interpretation of the SD Bioline results, for example, showed that the format of RDT impacted on the effectiveness of interpretation. Photographed RDTs helped to identify RDTs that are easiest to interpret for non-laboratory personnel, and this user-friendliness might provide additional insight to choose between two RDTs with similar laboratory performance. Last, photograph-based training could be extended to other media such as email, websites and mobile phones. This opens up possibilities such as RDT result confirmation by off-site proficient personnel and could offer on-going distance training in interpretation, thus maximizing the testing quality of diagnoses obtained using HIV RDTs.

The present study showed that a minimal training exercise can improve interpretation of HIV RDT, and that training materials can be useful in the format of photographed RDT results. Participants showed significant improvement in RDT interpretation, and photographed RDT assessed and identified that the types of result and participants that most benefited from the training. Therefore, photographed RDT can be a cost-effective way to assess, train and improve the interpretation proficiency of RDT operators, who might or might not have established proficiency in RDT operation and especially in those who have limited experience in interpretation.
